# A LONGITUDINAL STUDY ON SNAP PARTICIPATION AND SELF-REPORTED HEALTH AMONG DIABETIC AND NON-DIABETIC INDIVIDUALS

**DOI:** 10.1093/geroni/igad104.3141

**Published:** 2023-12-21

**Authors:** Danielle Duran, Nasim Ferdows

**Affiliations:** Northeastern University, Boston, Massachusetts, United States; Northeastern University, Boston, Massachusetts, United States

## Abstract

Self-reported health is a strong predictor of future health status, new morbidity, mobility decline, illness recovery, and healthcare utilization. Because self-reported health is rooted in social position and social comparison, it dually reflects individual health perceptions and health inequities, especially among those who experience a social struggle for resources. Food insecurity is associated with higher risk of chronic diseases and higher mortality rates. Interventions such as the supplemental nutrition assistance program (SNAP) were created to help address food insecurity. Although morbidity and mortality risks caused by food insecurity is exacerbated for those with nutrition-centered chronic disease, such as diabetes, limited research has assessed the impact of food insecurity and SNAP participation on health among diabetics. Using Health and Retirement Study data, we explore the effect of SNAP participation and food insecurity on the likelihood of reporting fair or poor health comparing 24,446 diabetes to 69,260 non-diabetics individuals aged 65 and above (2004-2020). Our analysis controls for key socioeconomic factors, including age, gender, race/ethnicity, and wealth, accounting for the complex design of HRS. Our key findings reveal that food insecure diabetics have significantly higher odds of reporting fair/poor health, and diabetic SNAP participants also had significantly higher odds of reporting fair/poor health. Our study demonstrates how food insecurity and diabetes concurrently influence health and how, despite the presence of resources, such as SNAP, there was no improvement in self-reported health. This study is important as it highlights the need for future research on the effects of SNAP participation and self-reported health.

